# A Systematic Comparison of Supervised Classifiers

**DOI:** 10.1371/journal.pone.0094137

**Published:** 2014-04-24

**Authors:** Diego Raphael Amancio, Cesar Henrique Comin, Dalcimar Casanova, Gonzalo Travieso, Odemir Martinez Bruno, Francisco Aparecido Rodrigues, Luciano da Fontoura Costa

**Affiliations:** 1 Institute of Mathematics and Computer Science, University of São Paulo, São Carlos, São Paulo, Brazil; 2 São Carlos Institute of Physics, University of São Paulo, São Carlos, São Paulo, Brazil; Shanghai Jiaotong University, China

## Abstract

Pattern recognition has been employed in a myriad of industrial, commercial and academic applications. Many techniques have been devised to tackle such a diversity of applications. Despite the long tradition of pattern recognition research, there is no technique that yields the best classification in all scenarios. Therefore, as many techniques as possible should be considered in high accuracy applications. Typical related works either focus on the performance of a given algorithm or compare various classification methods. In many occasions, however, researchers who are not experts in the field of machine learning have to deal with practical classification tasks without an in-depth knowledge about the underlying parameters. Actually, the adequate choice of classifiers and parameters in such practical circumstances constitutes a long-standing problem and is one of the subjects of the current paper. We carried out a performance study of nine well-known classifiers implemented in the Weka framework and compared the influence of the parameter configurations on the accuracy. The default configuration of parameters in Weka was found to provide near optimal performance for most cases, not including methods such as the support vector machine (SVM). In addition, the k-nearest neighbor method frequently allowed the best accuracy. In certain conditions, it was possible to improve the quality of SVM by more than 20% with respect to their default parameter configuration.

## Introduction

In the last decades, we have witnessed a progressive increase of data production and storage. Indeed, the informatization of most aspects of human activities, ranging from simple tasks such as phone calls to shopping habits, generates an ever increasing collection of data that can be organized and used for modeling and planning. At the same time, most scientific research projects – such as in genetics, astronomy and neuroscience – generate large amounts of data that needs to be analyzed and understood. This trend has given rise to new terms such as *big data* [1], [2]. Once such data is organized in a dataset, it is necessary to find patterns concealed in the vast mass of values, which is the objective of *data mining* [3]–[10]. Because the identification of important patterns (e.g. those that recur frequently or are rare) is impossible to be performed manually, it is necessary to resort to automated pattern recognition. Nevertheless, it is important to note that pattern recognition remains also relevant for organizing and understanding smaller sets of data, such as in medical diagnosis, industrial quality control, and expensive data.

The problem of pattern recognition consists in assigning classes or categories to observations or individuals [7], [8], [10]. This can be done in two main ways: (i) with the help of examples or prototypes (*supervised classification*); and (ii) taking into account only relationships between the properties of the objects (*unsupervised classification* or *clustering*). Though seemingly simple, pattern recognition often turns out to be a challenging activity. This is mainly a consequence of *overlap* between different groups in the data, i.e. objects in a class have similar properties as those in other classes. However, several other issues such as choice of features, noise, and sampling, also impose further problems while classifying data [7], [8], [10]. Even when the features are well-chosen and the data has good quality (e.g. properly sampled and without noise), the results of the classification will frequently vary with the choice of different pattern recognition methods and respective parameters. This situation is typically more critical for sparse data, presence of noise, or non-discriminative features. In an attempt to circumvent such problem and to obtain more robust and versatile classifiers, a number of pattern recognition methods have been proposed in the literature [11]–[13]. Yet, despite the long tradition of pattern recognition research [10], there are no definite guidelines for choosing classifiers. So, those faced with the need to apply pattern recognition are left with the difficult task of choosing among several alternative methods.

There are many works in the literature describing which classifiers are more suitable for specific tasks (see e.g. [14]–[16]), but only a few consider a more systematic quantitative analysis of their performance. Typical datasets employed to compare the performance of different methods include real world and/or artificial data. Advantages of using real datasets include the presence of non-trivial relationships between variables, which may strongly influence the performance of a classifier, the fact that the obtained results will usually be of high confidence when used for samples obtained in the same domain and using a similar criteria, and the presence of noise or unavailable information about the samples (hidden variables). But there is a main drawback associated with using real-world data. Even if one manages to consistently compare the results obtained with hundreds of real world datasets, the results still remain specific to the datasets being used. Trying to extend the information gained in such analyses to a different dataset can be ineffective. Furthermore, obtaining more real data to evaluate other classifier characteristics represents sometimes an arduous task. This is the case of applications whose data acquisition process is expensive. For these reasons, here we chose synthetic datasets. Although such datasets are often not representative of specific real-world systems, they can still be used as representations of large classes of data. For example, we can define that all variables in the dataset will have a given Pearson correlation, and study the behavior of the classifiers when setting this as the main data constrain. In addition, artificial datasets allow a systematic variation of respective parameters, and also provide exact ground truths. A natural choice of distribution for the variables in the dataset is the multivariate normal distribution. This choice is related to the central limit theorem [17], which states that, under certain conditions, the mean of a large number of independent random variables will converge to a normal distribution. All in all, we believe that the adoption of the multivariate normal density is capable of modeling a representative number of real cases. Nevertheless, we observe that the comparative performance of the methods present in this paper may change for other databases or conditions.

Since one of our main concerns is conducting an accessible practical study of the classifiers, we decided to consider the classifiers implemented by the Weka software [18], which is available at http://www.cs.waikato.ac.nz/ml/weka. In particular, we decided to use Weka because of its popularity among researchers. In addition, since the software is open-source, any researcher can check the code of any specific classifier. Since Weka includes many classifiers, we decided to select a subset of those most commonly used [19].

One distinctive feature of the present work is the procedure we use to compare classifiers. Many works in the literature try to find the best accuracy that a classifier can give and then present this value as the quality of the classifier. However, finding the highest accuracy for a classifier is usually not straightforward. Additionally, if this high accuracy can only be achieved for very specific data and values of the classifier parameters, it is likely that for a different dataset the result will be worse, since the parameters were tuned for the specific data analyzed. Therefore, besides giving a high accuracy, it is desirable that the classifier performs well without being *too sensitive* to parameter changes. That is, a good classifier should provide a robust classification for a reasonably large range of values of its parameters.

This work is organized as follows. We start by describing the generation of synthetic datasets and justifying its respective parameters. Next, we introduce the measurements used to quantify the classifiers performance. The comparative analysis involves the following three approaches. First, we compare the performance of the classifiers when using the default parameters set by Weka. This is probably the most common way researchers use the software. This happens because changing the classifier parameters in order to find the best classification value is a cumbersome task. Then, we address the variation of single parameters of the classifiers, while maintaining other parameters at their default values. That is, we study how the classification results are affected when changing each parameter, given that some parameters are more critical for the performance. Finally, in order to estimate the optimum accuracy of the classifier, as well as to verify its sensitivity to simultaneous changes of its parameters, we randomly sample the sets of parameter values to be used in the classifier.

### Related works

Typical works in the literature dealing with comparison between classifiers can be organized into two main groups: (a) comparing among a relatively few methods for the purpose of validation and justification of a new approach (e.g. [20]–[24]); and (b) systematic qualitative and quantitative comparison between many representative classifiers. Examples of qualitative analysis in (b) can be for example found in [19], [25], [26]. These studies perform a comprehensive analysis of several classifiers, describing the drawbacks and advantages of each method. A quantitative analysis of classifiers was performed in [27], where 491 papers comparing quantitatively at least two classification algorithms were analyzed.

Some studies in the literature are devoted to devising novel methodologies to analyze statistically the results obtained from the comparison of classifiers. The suitability of traditional measures was investigated in [28], which concluded that the simplest traditional accuracy indices should be preferred when the comparison is not focused on specific classes. Some studies show that many of the papers aiming at comparing the performance of different classifiers are limited in the sense that they compare several methods with respect to relatively few datasets [29], [30]. Most importantly, the investigation carried out in [31] warns that invalid conclusions can be drawn if specific statistical tests are not applied.

Many comparative studies are specific to a given problem or task. Perhaps this is a consequence of the “No Free Lunch theorem”, which states that, without any prior, no single method can be preferred [32]–[34]. A comparison of three representative learning methods (Naive Bayes, decision trees and SVM) was conducted in [35], concluding that Naive Bayes is significantly better than decision trees if the area under curve is employed as a performance measurement. Other quantitative studies compare, for example, artificial neural networks with other methods [36]. An extensive comparison of a large set of classifiers over many different datasets performed in [37] showed that SVMs perform well on classification tasks. Quantitative comparisons between classifiers can be also found in specific domain problems, such as in Bioinformatics [38], Computer Science [39]–[42], Medicine [43], [44], Civil Engineering [45] and Chemistry [46].

Some studies have investigated the influence of parameters on the performance of classifiers. In [47] the authors studied the sensitivity of parameters in accuracy-based learning systems, concluding that particular thresholds should be taken into account in order to prevent critical decreases in performance. A common topic related to the study of classifiers sensitivity concerns the optimization of parameters via heuristic methods. In [48] the authors propose a method to optimize both the parameter values and feature set for a SVM applied to the task of pedestrian detection. A general framework for the parameter selection problem employing Grid Search and Experiment Design is detailed in [49]. Although Grid Search strategies tend to yield better results, genetic methods can provide good results at a smaller computational cost. For this reason, some papers deal with the problem of optimizing the initial conditions of genetic algorithms to improve their accuracy [50]. Finally, parameter optimization has been studied in specific tasks, such as in biological and textual applications [51], [52].

## Materials and Methods

In this section we present a generic methodology to construct artificial datasets modeling the different characteristics of real data. In addition, we describe the measurements used to evaluate the quality of the classifiers.

### Artificial Data

Here we present an adapted method for generating random datasets with a given ensemble of covariance matrices, which was based on the study made by Hirschberger et al. [54]. We aim at generating 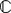 classes of data with 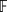 features for each object, with the additional constraint that the number of objects per class is given by the vector 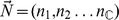. This problem is mathematically restated as finding 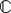 sets comprising 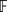-dimensional vectors, where each set has a number of elements specified by 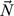. Furthermore, we aimed at generating data complying with the three following constraints:

- **Constraint 1**: the variance of the 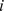-th feature of each class is drawn from a fixed distribution, 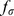.
- **Constraint 2**: the correlation between the 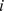-th and 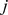-th dimension of each class are drawn from another fixed distribution, 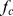.
- **Constraint 3**: we can freely tune the expected separation between the classes, given by parameter 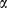, which is explained below.

Traditionally, constraints 1 and 2 are not fully satisfied to generate the data. Many studies impose that all the classes display approximately the same variances and correlations, by defining an ensemble of covariance matrices with a fixed spectrum constraint [55], [56]. Unfortunately, this approach is somewhat artificial to generate realistic data, since the assumption that all data classes share similar relationships between their features is quite unlikely. Our approach is more general because, given the shape of the correlation distribution (e.g. U-shaped), the classes can exhibit all kinds of correlations.

In order to generate the data with the parameters 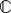, 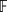 and 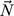 complying with constraints 1, 2 and 3, we need 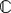 covariance matrices (one for each class), where each diagonal and off-diagonal element is drawn, respectively, from 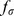 and 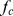. The most common approach is to randomly draw the mentioned matrix elements from probability density distributions given by 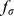 and 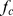 in order to construct the desired matrices. Unfortunately, this process does not guarantee a valid covariance matrix because every covariance matrix must be positive and semi-definite [57]. To overcome this problem we use a well-known property stating that for every matrix 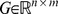, the 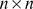 matrix 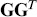 is positive and semi-definite [57]. This property allows us to create a random matrix 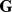 that will generate a valid covariance matrix. The matrix 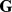 is known as *root* matrix. Next, it is necessary to define a convenient root matrix so that 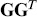 follows constraints 1, 2 and 3. Hirschberger et al. [54] came up with an elegant demonstration on how to create a covariance matrix following constraints 1 and 2. Using their algorithm, modified in order to specify the separation between the classes (parameter 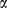), it is possible to create datasets having the following parameters:

- **Number of objects per class** 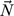: The number of instances in each class can be drawn according to a given distribution. The most common distributions to use are the normal, power-law and exponential distributions. Nevertheless, in order to simplify our analysis, here we use classes having an equal number of instances, 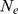, which varies from 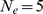 to 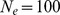 elements.
- **Number of classes** 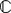: This parameter is varied from 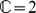 to 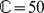.
- **Number of features** 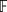: The case 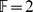 represents the simplest case, since it permits the easy visualization of the data. In order to improve the discriminability of the data, real world datasets oftentimes are described by a larger number of features. Here we vary 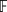 in the range [2], [50]. Hereafter, we refer to the dataset described by 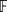 features as DB
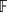
F.
- **Standard deviation of the features**: For each class, the standard deviation of each feature is drawn according to a given distribution 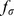. The process is repeated for each class, using the same distribution 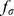.
- **Correlation between features**: For each class, the correlations between the features are drawn according to a given distribution 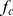. The process is repeated for each class using the same distribution. This means that each class of our dataset will show different values of correlation. For example, instances from one class may be described by redundant features, while the same features may be much more efficient in describing samples from other classes. The most common choices for 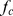 are: (a) *uniform*, to represent heterogeneous data; (b) zero mean *normal*, for mostly uncorrelated data; and (c) *U-shaped*, for data with strong correlations. Here we chose a uniform distribution for the correlations.
- **Separation between the data (** 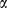 **)**: It is a parameter to be varied throughout the experiments, quantifying how well-separated are the classes, compared to their standard deviation. This parameter is simply a scaling of the standard deviation of the features for each class, i.e., the values drawn from the distribution 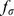 are divided by 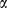. Since we randomly draw the mean, 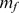, for each class in the range 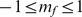, 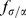 can be used to define an expected separation between the classes. If 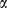 is large, the classes are well-localized and will present little overlap. Otherwise, if 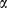 is small, the opposite will happen. Clearly, the separation given by 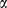 depends on the dimension of the space. Nevertheless, there is no need to define a normalization for 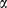, because we are just comparing classifiers and not different configurations of the data.

**Figure 1:**
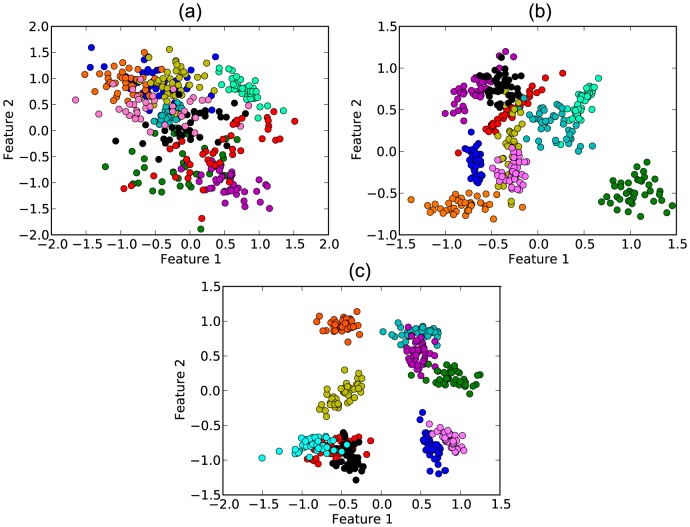
Example of artificial dataset for 10 classes and 2 features (DB2F). It is possible to note that different classes have different correlations between the features. The separation between the classes are (a) 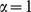, (b) 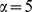 and (c) 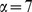.

In Figure 1 we show some examples of the data that can be generated by varying 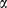 in a two-dimensional dataset.

### Evaluating the performance of the classifiers

A fundamental aspect that should be considered when comparing the performance of classifiers is the proper definition of what *quality* means. It is impossible to define a single metric that will provide a fair comparison in all possible situations. This means that quality is usually specific to the application and, consequently, many measurements have been proposed [53]. Nevertheless, there are some measurements that have widespread use in the literature, the most popular being the accuracy rate, f-measure (sometimes together with precision and recall), Kappa statistic, ROC area under curve and the time spent for classification (see [53] for a comprehensive explanation of such measurements). Because we are mostly interested in a more practical analysis of the classifiers, we use only the accuracy rate, which is defined as the number of true positives plus the number of true negatives, divided by the total number of instances.

To measure the performance of the classifiers, we generate artificial datasets using the method presented in the previous section and calculate some statistics. The principal quantity extracted from each dataset is the average accuracy rate. In addition, we also compute the variation of accuracy across datasets, as this quantity is useful to quantify the confidence of the classifier when the dataset is changed. The standard deviation of accuracy rate computed over instantiations of the classifier with distinct parameters is useful to quantify the sensitivity with respect to a given parameter.

## Results and Discussion

The performance of the classifiers was evaluated according to three methodologies. The default values provided by Weka were used first. We then examined the influence of each classifier parameter on the discriminability of the data. Finally, we study the performance when all classifier parameters are varied jointly. The classifiers considered in the analysis are presented in Table 1.

**Table 1:** List of classifiers employed in the analysis.

Type	Classifier name	Name in Weka
Bayesian	Naive Bayes	bayes.NaiveBayes
	Bayesian Network (Bayes Net)	bayes.net
Tree	C4.5	trees.J48
	Random Forest	trees.RandomForest
	Simple Classification and Regression Tree (CART)	trees.SimpleCart
Lazy	k-Nearest Neighbors (kNN)	lazy.IBk
Function	Logistic	functions.Logistic
	Multilayer Perceptron	functions.MultilayerPerceptron
	Support Vector Machine (SVM)	functions.SMO

List of classifiers evaluated in our study. The abbreviated names used for some classifiers are indicated after the respective name.

### Comparison of classifiers using their default parameters

The default values of the classifiers are often adopted by non-expert users, and provide a logical starting point for expert researchers. In order to provide a comprehensive comparison between classifiers, we employed the following parameters on the aforementioned algorithm to create the artificial datasets. The number of classes take the values 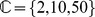, the number of features are 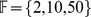 and the number of elements for each class is 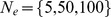. One possible drawback of studying such distinct scenarios is that depending on the value of the separation, 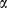, between the classes the accuracy of the classifiers might “saturate”. For example, when increasing the number of features, if the values of 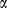 remains fixed, we expect all classifiers to provide accuracies close to 100%. Therefore, for each combination of the parameters 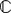, 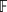 and 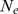 we performed a grid search to find the value 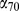 of 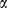 such that the accuracy rate of the Naive Bayes classifier is as close as possible to 70%. The idea behind this procedure is that fixing the accuracy rate of one classifier will likely avoid other classifiers from reaching too extreme values. The Naive Bayes classifier has been chosen because, during the experiments, it provided an accuracy close to the mean accuracy of all classifiers. In order to explore the behavior of the classifiers for accuracies close to 70%, but also to study what happens for larger or smaller class separations, we generated datasets taking the following values of class separability: 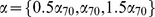. Considering all four parameter combinations, we have a total of 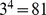 different parameter configurations. For each configuration, we take the mean value of 10 generated datasets.

The performance of each classifier over the 810 datasets, considering Weka's default parameters, is summarized in Tables 2 and 3. In Table 2, we show the percentage of parameter configurations where the classifier in row 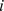 outperformed the classifier in column 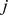. The last column shows the percentage of configurations where the classifier reached the best rank. It is clear that the Multilayer Perceptron classifier outperformed the Bayesian Network, C4.5, Simple CART and SVM when considering the default parameters. Surprisingly, the Multilayer Perceptron was only outperformed by the kNN, which is a much simpler and faster classifier. Actually, on average the kNN outperformed *all* classifiers, even cutting-edge classifiers like SVM and C4.5. Another classifier known for its simplicity, the Naive Bayes, also provided interesting results, showing an almost equal or better ranking than all other classifiers besides the Multilayer Perceptron. Since it is widely known that the Multilayer Perceptron usually requires a longer execution time, these results indicate that if the researcher has no a priori knowledge of the classifier parameters, the Naive Bayes and kNN classifiers could represent a good all-around option for using on his data. Nevertheless, it is also important to note that one reason that cutting-edge classifiers might not provide good results for the default parameters is that they are conceived to be versatile enough to be able to adapt to specific properties of the dataset. Therefore, their parameters can be optimized to the task at hand by using methods like grid search or simulated annealing. The drawback is that such optimization is not always straightforward, and needs an adequate training dataset.

**Table 2:** Relative rank of classifiers.

	Perceptron	kNN	Random Forest	Naive	Logistic	SVM	Bayes Net	C4.5	Simple CART	Top
Perceptron	-	35%	72%	62%	70%	91%	98%	93%	93%	32%
kNN	65%	-	78%	75%	60%	88%	91%	83%	79%	17%
Random Forest	28%	22%	-	41%	32%	80%	85%	78%	85%	17%
Naive Bayes	38%	25%	59%	-	60%	81%	95%	88%	96%	16%
Logistic	30%	40%	68%	38%	-	67%	86%	72%	75%	14%
SVM	7%	12%	20%	19%	33%	-	47%	26%	37%	2%
Bayes Net	2%	9%	15%	5%	14%	53%	-	22%	33%	1%
C4.5	6%	17%	22%	12%	28%	74%	78%	-	80%	0%
Simple CART	7%	21%	15%	4%	25%	63%	67%	20%	-	0%

Percentage of parameter configurations where the classifier in row 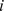 had an higher accuracy than the classifier in column 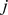. The last column shows the percentage of configurations in which each respective classifier provided the highest accuracy.

**Table 3:** Difference in mean accuracy of classifiers.

	Perceptron	kNN	Random Forest	Naive	Logistic	SVM	Bayes Net	C4.5	Simple CART	M Acc
Perceptron	-	−8.0%	2.4%	2.4%	5.5%	19.8%	20.6%	13.0%	17.1%	74.3%
kNN	8.0	-	10.4%	10.4%	13.5%	27.8%	28.6%	20.9%	25.1%	82.2%
Random Forest	−2.4%	−10.4%	-	0.0	3.1%	17.4%	18.2%	10.5%	14.7%	71.8%
Naive Bayes	−2.4%	−10.4%	0%	-	3.1%	17.4%	18.2%	10.5%	14.7%	71.8%
Logistic	−5.5%	−13.5%	−3.1%	−3.1%	-	14.3%	15.1%	7.4%	11.6%	68.7%
SVM	−19.8%	−27.8%	−17.4%	−17.4%	−14.3%	-	0.7%	−6.9%	−2.8%	54.4%
Bayes Net	−20.6%	−28.6%	−18.2%	−18.2%	−15.1%	−0.7%	-	−7.6%	−3.5%	53.7%
C4.5	−13.0%	−20.9%	−10.5%	−10.5%	−7.4%	6.9%	7.6%	-	4.1%	61.3%
Simple CART	−17.1%	−25.1%	−14.7%	−14.7%	−11.6%	2.8	3.5%	−4.1%	-	57.2%

Mean difference between the accuracy of classifier in row 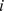 and the classifier in column 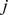. The last column shows the mean accuracy of the respective classifier for all datasets considered in our study.

The relative rankings of the classifiers provide concise information about the fraction of times a classifier can be considered better than the others. Nevertheless, an additional information needed to provide a fairer comparison of classifiers is the difference of their mean accuracies. This is necessary because the relative ranking indicates whether the accuracy of a classifier is higher than the other, but not *how large* the difference in accuracy is. In Table 3 we show the mean difference in accuracy between the classifiers for the 81 parameter configurations. Also shown on the last column is the mean accuracy of each classifier over all configurations. By comparing Tables 2 and 3 it is clear that although the Multilayer Perceptron usually outperforms other classifiers, it is not always by a large margin. For example, although the Multilayer Perceptron is 72% of the time better ranked than the Random Forest, on average the improvement is only 2.4%. Another interesting result is that the kNN provided the highest mean accuracy. Comparing the kNN with the Multilayer Perceptron the situation is as follows. The kNN usually provides a larger accuracy than the Multilayer Perceptron, but in cases where the Multilayer Perceptron is better than the kNN, it becomes the best classifier. This means that in the scenario where the default parameters are to be used, when the accuracy given by the kNN is not satisfactory, it may be worth using the Multilayer Perceptron instead.

**Figure 2:**
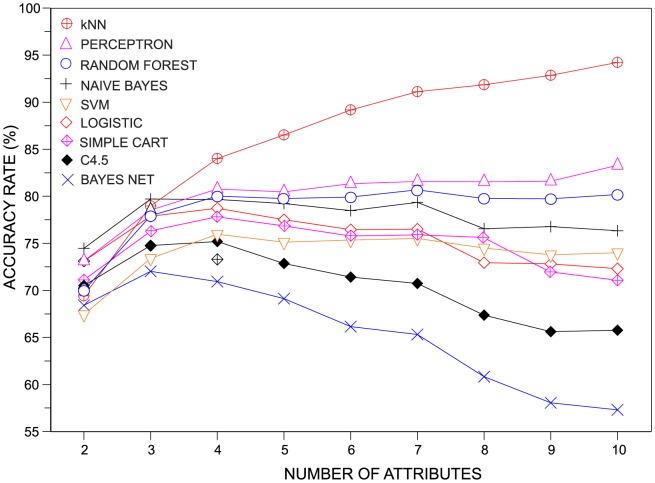
Behavior of the accuracy rate as the number of features increases. As more attributes are taken into account, the kNN becomes significantly better than the other pattern recognition techniques.

Another interesting issue to be investigated concerns the assessment of the discriminability of the classifiers as the number of features increases continually from two to ten features. To perform this analysis, we assessed the accuracy of the classifiers in datasets having 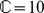, 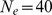 and 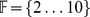. The increase in the number of features tends to result in higher accuracies. Therefore, in order to avoid a trivial result of all classifiers providing better results when including more features, for each dataset we set 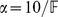. In Figure 2 we show the variation of the average accuracy as the number of features describing the dataset is incremented. Three distinct behaviors can be clearly observed: (i) the accuracy increases; (ii) the accuracy is nearly constant; and (iii) the accuracy decreases. The only classifier in which the pattern (i) was observed was the kNN classifier. The behavior (ii) is the most common trend. Finally, the third effect is more prominent in the case of the C4.5, Simple CART and Bayesian Network. All in all, these results suggest that the kNN performs significantly better than others classifiers in higher (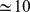) dimensions.

### Varying parameters: One-dimensional analysis

An alternative scenario in typical classification tasks arises when the researcher or practitioner wants to improve the performance of the classification by changing the values of the parameters. In this case, we turn to the concept of *sensitivity* of the classification with regard to the parameters. In other words, if a good classification is achieved only for a very small range in the parameter space, then for many applications it will be very difficult to achieve the best accuracy rate provided by the classifier. Conversely, if the classifier provides high accuracy rates for many different configuration of parameters, then one expects that it will consistently yield high-quality classifications irrespectively to the chosen parameters.

**Figure 3:**
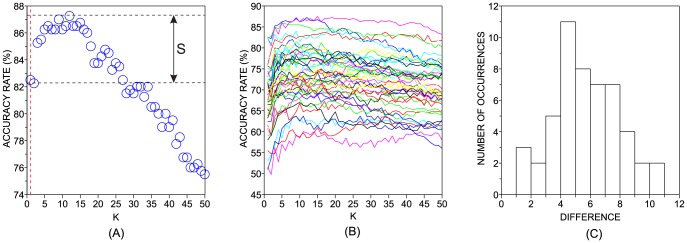
One dimensional analysis performed with the parameter 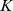
 **of the kNN classifier.** Panel (a) illustrates the default value of the parameter (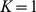) with a red vertical dashed line. The accuracy rate associated with default values of parameters is denoted by 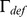 and the best accuracy rate observed in the neighborhood of the default value of 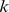 is represented as 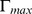. The difference between these two quantities is represented by 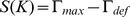. Panel (b) shows how the accuracy rates vary with the variation of 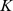 in DB2F (each line represent the behavior of a particular dataset in DB2F). Finally, panel (c) displays the distribution of 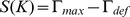 in DB2F.

To probe the sensitivity of the classifiers with regard to distinct values or parameters, we analyzed the behavior of the accuracy rate curves when each parameter is varied separately while keeping the remaining parameters set at their default values. This one-dimensional analysis is illustrated in Figure 3. Since the analysis of varying the classifiers parameters for all 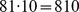 generated datasets would be impossible, here we use only two datasets. Both datasets have 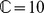 and 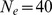, the dataset we call DB2F has 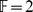 and 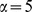, and the dataset called DB10F has 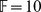 and 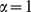. The behavior of the accuracy with the adoption of values different from the default for some parameters is shown in Figures S1 and S2 in File S1. For the sake of completeness, tables S1–S3 in File S1 show the accuracy rates obtained for the case where default parameters were employed in the same database.

An extensive analysis comparing the quality achieved with the parameters set with default and non-default values is provided in Table 3 for classifications obtained in DB2F. For each parameter, we provide the average 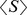, the standard deviation 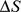 and the maximum value of 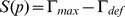, where 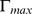 and 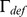 are respectively the maximum accuracy observed when the parameter 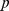 varies and the accuracy rate obtained with all the parameters set at their default values. Therefore, the statistic computed over 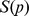 quantifies how much the accuracy of the classification is influenced when each parameter is set to a value different from the default. Table 3 shows that 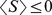 for almost all parameters, with the only exceptions being the number of seeds (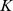) of the kNN and the type (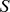) of SVM used. This result suggests that the default parameters usually provide a classification performance that is close to the optimum. Interestingly, even the maximum gain in accuracy is usually small, as they do not exceed 6.25% in any particular dataset (aside from the kNN and SVM classifiers).

Similarly to Table 4, Table 5 shows the results for single parameter variation for classifications performed in DB10. We note that a proper analysis of this table must consider the accuracy rate obtained with default parameters (see Table S2 in File S1), because the latter has a large variation across classifiers. Therefore, if a classifier performs very well with parameters set with their default values, one expects that a significant improvement through one-dimensional variation of parameters will be less probable. This effect becomes evident when one analyzes, for example, the kNN. The default configuration of parameters yields high accuracy rates (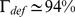), while the average improvement through one-dimensional analysis is only 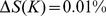. A significant improvement in the discriminability was observed for the Multilayer Perceptron through the variation of the size of the hidden layers (H). In a similar manner, a significant increase of accuracy was observed when we varied the number of trees (I) of the Random Forest. As for the SVM classifier, six of its parameters allowed an increase of about 20%, which led to accuracy rates higher than 94% in many cases. This result suggests that appropriate parameter tuning in SVM might improve significantly its discriminability.

**Table 4:** One-dimensional analysis of parameters performed in DB2F.

Classifier	Parameter	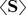 (%)	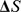 (%)	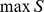 (%)
Bayes Net	-D	0.00	0.00	0.00
kNN	-K	6.62	2.45	12.75
kNN	-I	0.00	0.00	0.00
kNN	-F	0.00	0.00	0.00
kNN	-X	0.00	0.00	0.00
C4.5	-U	−0.18	0.72	1.25
C4.5	-S	0.04	0.26	1.00
C4.5	-A	0.00	0.00	0.00
C4.5	-C	0.69	0.54	2.00
C4.5	-M	0.86	0.76	2.75
C4.5	-N	0.23	1.36	2.75
Logistic	-R	0.63	0.60	2.25
Logistic	-M	0.84	0.61	2.75
Nave Bayes	-K	−0.74	1.15	1.75
Nave Bayes	-D	−5.79	3.64	1.25
Perceptron	-D	−51.25	7.17	−37.75
Perceptron	-C	0.00	0.00	0.00
Perceptron	-H	1.74	1.61	6.25
Perceptron	-L	1.30	0.88	3.75
Perceptron	-M	1.17	0.83	3.75
Perceptron	-N	1.00	0.66	3.00
Perceptron	-V	0.74	0.75	2.50
Perceptron	-E	0.00	0.00	0.00
Random Forest	-I	0.02	0.14	1.00
Random Forest	-K	−0.09	0.64	−4.50
Random Forest	-depth	0.03	0.18	1.25
Random Forest	-S	0.04	0.28	2.00
Simple CART	-S	0.06	0.39	2.75
Simple CART	-C	0.00	0.00	0.00
Simple CART	-M	0.04	0.25	1.75
Simple CART	-N	0.02	0.11	0.75
Simple CART	-A	0.01	0.07	0.50
Simple CART	-H	0.00	0.00	0.00
Simple CART	-U	−0.01	0.07	−0.5
SVM	-C	0.05	0.32	2.25
SVM	-L	0.01	0.07	0.50
SVM	-P	0.03	0.21	1.50
SVM	-V	0.00	0.00	0.00
SVM	-N	0.03	0.21	1.50
SVM (poly kernel)	-E	1.38	1.29	4.50
SVM (NP kernel)	-E	−20.87	5.28	−8.00
SVM (RBF kernel)	-G	2.55	2.55	12.75
SVM (Puk kernel)	-S	5.88	2.46	11.75

Comparison between the accuracy achieved with the default and the best parameter. The difference between the former and the latter in DB2F was summarized with the average, the standard deviation and the maximum difference. Note that for most of the parameters the average 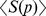 is not significantly greater than zero, suggesting that default parameters provide a good discriminability of the data.

**Table 5:** One-dimensional analysis of parameters performed in DB10F.

Classifier	Parameter	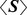 (%)	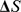 (%)	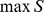 (%)
Bayes Net	-D	0.00	0.00	0.00
kNN	-K	0.01	0.04	0.25
kNN	-I	0.00	0.00	0.00
kNN	-F	0.00	0.00	0.00
kNN	-X	0.00	0.00	0.00
C4.5	-U	−0.05	0.29	0.75
C4.5	-S	−0.01	0.13	0.25
C4.5	-A	0.00	0.00	0.00
C4.5	-C	0.27	0.30	1.25
C4.5	-M	1.32	0.96	3.50
C4.5	-N	−7.44	1.75	−2.75
Logistic	-R	0.58	0.71	4.25
Logistic	-M	0.81	0.73	4.25
Naive Bayes	-K	−2.91	1.64	1.25
Naive Bayes	-D	−19.20	3.10	−11.75
Perceptron	-D	−56.10	5.33	−46.50
Perceptron	-C	0.00	0.00	0.00
Perceptron	-H	7.06	2.53	13.25
Perceptron	-L	2.27	1.11	5.50
Perceptron	-M	2.33	1.00	4.25
Perceptron	-N	1.01	0.77	4.00
Perceptron	-V	0.45	0.76	2.75
Perceptron	-E	0.00	0.00	0.00
Random Forest	-I	5.67	1.73	10.50
Random Forest	-K	0.54	0.98	3.75
Random Forest	-depth	1.11	1.03	3.75
Random Forest	-S	3.04	1.86	8.75
Simple CART	-S	1.09	0.81	2.75
Simple CART	-C	0.00	0.00	0.00
Simple CART	-M	1.41	1.16	4.25
Simple CART	-N	1.31	0.92	3.25
Simple CART	-A	3.89	1.70	9.00
Simple CART	-H	0.00	0.00	0.00
Simple CART	-U	−1.21	1.18	−4.00
SVM	-C	22.15	4.10	36.50
SVM	-W	0.38	0.33	1.50
SVM	-P	0.52	0.67	3.25
SVM	-V	0.00	0.00	0.00
SVM	-N	23.54	4.60	39.30
SVM (poly kernel)	-E	23.57	4.40	37.75
SVM (NP kernel)	-E	19.08	4.84	31.00
SVM (RBF kernel)	-G	21.55	3.91	34.75
SVM (Puk kernel)	-S	19.90	3.59	32.50

Comparison between the accuracy achieved with the default and the best parameter. The difference between the former and the latter in DB10F was summarized with the average, the standard deviation and the maximum difference. The appropriate tuning of C, N, E, G and S can improve significantly the performance of the default SVM.

### Varying parameters: Multidimensional analysis

**Figure 4:**
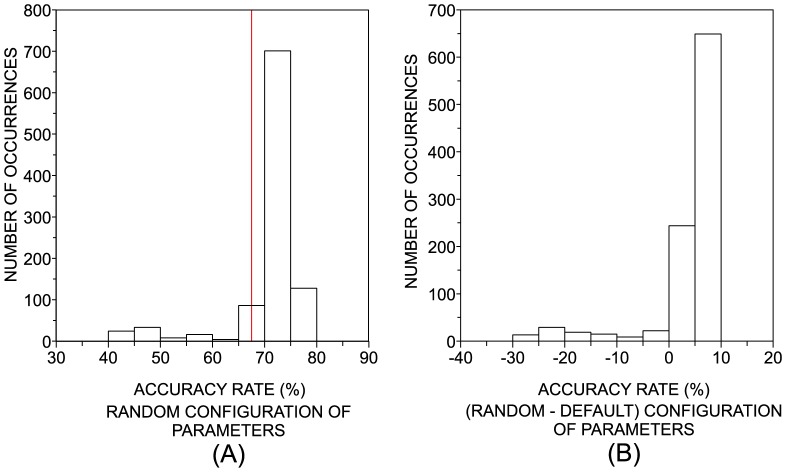
Example of the random parameters analysis. We use one of the artificial datasets and the kNN classifier. (a) By randomly drawing 1,000 different parameter combinations of kNN we construct a histogram of accuracy rates. The red dashed line indicates the performance achieved with default parameters. (b) The accuracy rate for the default parameters are subtracted from the values obtained for the random drawing. The normalized area of the histogram for values that are above zero indicates how easy is to improve the performance with a random tuning of parameters.

Although the one-dimensional analysis is useful to provide relevant information regarding the variability of accuracy with regard to a given parameter, this type of analysis deliberately disregards the influence of possible mutual interdependencies among parameters on the performance of the classifiers. In order to consider this interdependence, we randomly sample the values of parameters in a bounded range. More specifically, 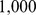 random configurations of parameters for each classifier were generated and each classifier was applied to discriminate the classes in DB2F and DB10F. Note that the Naive Bayes and Bayesian Net classifiers were not included in the multidimensional analysis, since they only have binary parameters. In the next step, we compared the performance of the best random configuration with the performance achieved with the default parameters. An example of the procedures adopted in the multidimensional analysis is provided in Figure 4. A more ‘efficient’ possibility could be based on the search of the best accuracy rates (considering all configuration of parameters) through an optimization heuristic. Nevertheless, we decided to avoid optimization heuristics because this would imply many problems caused by the different kinds of parameters used in distinct classifiers (e.g., nominal, binary, integer, etc). Moreover, it would be difficult to avoid local extremes which are typical of such approaches.

**Figure 5:**
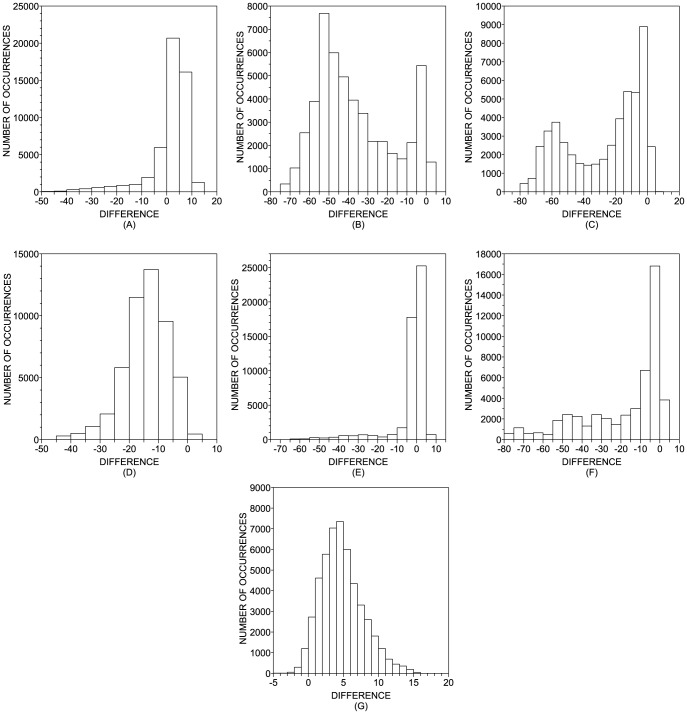
Distribution of the difference of accuracy rates observed between the random and default configuration of parameters. (a) kNN; (b) C4.5; (c) Multilayer Perceptron; (d) Logistic; (e) Random Forest; (f) Simple CART; (g) SVM. Note that, in the case of kNN and SVM classifiers, most of the random configurations yield better results than the default case.

In Figure 5, we show the histograms of the accuracy rates obtained with the random choice of parameters of the classifiers, which were evaluated in DB2F. In order to summarize the main characteristics observed in these histograms, in Table 6 we show some statistics taken over the histograms. The 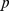-value quantifies the percentage of realizations in which a random configuration of parameters improved the performance obtained with the default configuration. Considering the cases where an improvement was observed, we can summarize the values of accuracy in terms of their average, standard deviation and maximum value. It is noteworthy that the random choice of parameters usually reduces the accuracy (i.e. 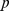-value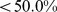) for Simple CART, Multilayer Perceptron, C4.5 and Logistic. This means that one should be aware when choosing parameters other than the default configuration, since most of the random configurations impact the performance negatively. Surprisingly, in almost every random choice of parameters (96.89% of the cases) the accuracy of the SVM increases. In the case of the kNN, the improvement is less likely (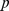-value = 76.15%). The Random Forest shows a typical small improvement in 52% of the realizations, in comparison with SVM and kNN. Whenever the computing time for each dataset is not very high, it is possible to generate many random configurations and select that providing the highest accuracy. In this case, the most important parameter extracted from Table 6 becomes the maximum accuracy. This scenario is particularly useful for SVM, kNN and Random Forest, since the performance can be improved in 16%, 13% and 10%, respectively. Actually, SVM and kNN emerge as the best classifiers when we consider only the best realization among the 1,000 random configurations for each dataset (see Table 7).

**Table 6:** Multidimensional analysis of parameters performed in DB2F.

#	Classifier	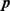 -value	Mean (%)	Deviation (%)	Maximum (%)
**1**	SVM	96.89	5.07	2.87	16.00
**2**	kNN	76.15	4.90	2.54	13.00
**3**	Random Forest	51.93	1.82	1.34	10.25
**4**	Simple CART	7.68	0.91	0.57	3.75
**5**	Multilayer Perceptron	4.87	1.28	1.00	6.75
**6**	C4.5	2.56	0.93	0.69	3.50
**7**	Logistic	0.88	0.77	0.48	2.75

Comparison of classifiers using a multidimensional analysis of classifiers evaluated in DB2F. 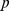-value represents the percentage of cases where the random configuration of parameters yields a classifiers that outperforms the classifier obtained with default parameters. Mean, deviation and maximum refer to the increase in accuracy provided by the random configuration, when it outperforms the default configuration. In the case of the SVM, the random choice of parameters yields a classification more accurate than the default classification in about 97% of the cases. In this case, the average and maximum improvement of quality are 5% and 16%, respectively.

**Table 7:** Ranking of classifiers in DB2F.

#	Classifier	Average (%)	Deviation (%)
**1**	SVM	78.1	5.0
**2**	kNN	75.9	6.2
**3**	Multilayer Perceptron	75.4	6.2
**4**	Random Forest	77.3	5.1
**5**	Logistic	73.5	6.0
**6**	Simple CART	72.8	6.3
**7**	C4.5	71.5	6.5

Ranking of classifiers in DB2F considering the best configuration of parameters among the 1,000 random configurations.

Repeating the above analysis for the classifications performed in DB10F, one observes some differences in the results, which are shown in Table 8. From the analysis of the means (third column), it is clear that, apart from SVM, a significant improvement in accuracy is much less likely. These results reinforce the premise that default parameters generally provide an accuracy that is near to the optimum. However, as we found for DB2F, the performance of SVM can be significantly improved by the suitable configuration of parameters. Note that the average improvement of 20.35% is equivalent to that found with a one-dimensional variation in the complexity parameter (see parameter C in Table 5). Therefore, the best configuration of the SVM can be achieved by varying only one parameter. Again, if we consider only the best configuration among the 1,000 random configurations for each dataset, the SVM and kNN performs better than the other methods (see Table 9).

**Table 8:** Multidimensional analysis of parameters performed in DB10F.

#	Classifier	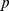 -value	Mean (%)	Deviation (%)	Maximum (%)
**1**	SVM	99.43	20.35	5.67	39.00
**3**	Random Forest	48.74	3.91	2.25	14.5
**2**	kNN	21.84	0.29	0.10	0.75
**4**	Simple CART	4.95	1.89	1.28	7.25
**5**	Multilayer Perceptron	4.11	3.25	2.46	12.00
**7**	Logistic	1.27	0.76	0.48	3.75
**6**	C4.5	0.47	1.23	0.90	3.50

Comparison of classifiers using a multidimensional analysis of classifiers evaluated in DB10F. 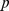-value represents the percentage of cases where the random configuration of parameters yields a classification that outperforms the classification obtained with default parameters. Mean, deviation and maximum refer to the increase in accuracy provided by the random configuration, when it outperforms the default configuration. In the case of the SVM, the random choice of parameters yields a classification more accurate than the default classification in about 99% of the cases. When this scenario occurs, the average and maximum improvement of quality are 20% and 39%, respectively.

**Table 9:** Ranking of classifiers in DB10F.

#	Classifier	Average (%)	Deviation (%)
**1**	SVM	98.8	0.7
**2**	kNN	94.3	1.8
**3**	Random Forest	88.7	1.9
**4**	Logistic	72.4	4.7
**5**	C4.5	67.1	2.8
**6**	Simple CART	66.3	3.5
**7**	Multilayer Perceptron	50.9	2.3

Ranking of classifiers in DB10F considering the best configuration of parameters among the 1,000 random configurations.

## Conclusions

Machine learning methods have been applied to recognize patterns and classify instances in a wide variety of applications. Currently, several researchers/practitioners with varying degrees of expertise have employed computational tools such as Weka to study particular problems. Since the appropriate choice of parameters requires certain knowledge of the underlying mechanisms behind the algorithms, oftentimes these methods are applied with their default configuration of parameters. Using the Weka software, we evaluated the performance of classifiers using distinct configurations of parameters in order to verify whether it is feasible to improve their performance. We should highlight here that results obtained from the comparison of different classification methods on the artificial dataset are not universal. This means that, in particular cases, the rank of classifiers may assume different configurations for distinct datasets, as it is widely known that machine learning methods are case-based learning. Nevertheless, we believe that our multivariate normal dataset encompasses a wide variety of real problems.

The analysis of performance with default parameters in the artificial dataset revealed that the kNN usually outperforms the other methods. The Multilayer Perceptron performed better than Bayesian Network, C4.5, SVM and Simple CART in most datasets. Unfortunately, this average gain is not significative and could not be justifiable in practical applications as the computational cost of the Multilayer Perceptron is higher than the cost of the other methods. The Naive Bayes also outperformed the Bayesian Network, C4.5, SVM and Simple CART. Surprisingly, the SVM implemented by Weka displayed an overall performance lower than the other methods when default parameters were employed in the analysis. When just one parameter was allowed to vary, there was not a large variation in the accuracy compared with the classification achieved with default parameters. The only exceptions were the parameter K of the kNN and parameter S of SVM (with Puk kernel). In these cases, the appropriate choice of the parameters enabled an average increase of 6% in accuracy. Surprisingly, we found that when the same analysis is performed with a ten-dimensional dataset, the improvement in performance surpasses 20% for the SVM. Finally, we developed a strategy in which all the configuration of parameters are chosen at random. Despite its outward simplicity, this strategy is useful to optimize SVM performance especially in higher dimensions (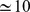), since the average increase provided by this strategy is higher than 20%.

Another important result arising from the experiments is the strong influence of the number of features on the performance of the classifiers. While small differences across distinct classifiers were found in 2–3 dimensions, we observed significant differences in performance when increasing the number of features. When assessing the performance of the classifiers in higher dimensions, kNN and SVM turned out to be the most accurate techniques when default and alternative parameters were considered, respectively. In addition, we found that the behavior of the performance with the number of features follows three distinct patterns (for the considered interval): (i) almost constant (Multilayer Perceptron); (ii) monotonic increase (kNN), and (iii) monotonic decrease (Bayesian Network). These results suggest that the number of features of the problem plays a key role on the choice of algorithms and, therefore, it should be considered in practical applications. In addition, for low dimension classification tasks, Weka's default parameters provided accuracy rates close to the optimal value, with a few exceptions. The highest discrepancies occurred when the performance of the SVM was assessed in higher dimensions, suggesting additional care while choosing the parameters in these conditions.

It is possible to perform further research to probe the properties of classifiers with regard to other factors such as number of classes, number of instances per class and overlap between classes. It is also important to probe the performance of supervised classifiers in problems where the number of training instances is limited because of their cost.

## Supporting Information

File S1 (PDF)
