# Supplementary material for: A Systematic Comparison of Supervised Classifiers
Source: PLoS One. 2014 Apr 24;9(4):e94137. doi: 10.1371/journal.pone.0094137 (PMC3998948; doi:10.1371/journal.pone.0094137)
Supplement: File S1 — (PDF) [file pone.0094137.s001.pdf]

A Systematic Comparison of Supervised Classifiers - Supporting Information

Diego Raphael Amancio[1,*], Cesar Henrique Comin[2], Dalcimar Casanova[2], Gonzalo Travieso[2], Odemir Martinez Bruno[2], Francisco Aparecido Rodrigues[1] and Luciano da Fontoura Costa[2]

1 Institute of Mathematics and Computer Science, University of São Paulo
São Carlos, São Paulo, Brazil

2 São Carlos Institute of Physics, University of São Paulo
São Carlos, São Paulo, Brazil

E-mail: diego.raphael@gmail.com, diego.amancio@usp.br

1. Classifiers

In the current investigation, we compared the performance of nine well-known classifiers (see Ref. [1]), which are available in the WEKA framework. Following the hierarchy defined in Ref. [2], these classifiers can be divided into the following groups: Bayesian an lazy classifiers, trees, and functions.

1.1. Bayesian classifiers

Bayesian classifiers assign membership probabilities to classify new objects. The method is fast and is known to yield fairly good results [3], even when applied to large volumes of data. The two bayesian classifiers, namely Naive Bayes and Bayesian Network, are described below.

1.1.1. **Bayesian Network:** A Bayesian Network model [4] is a probabilistic graph model (a type of statistical model) that represents a set of random variables and their conditional dependencies via a directed acyclic graph (DAG). The nodes represent random variables and the edges represent conditional dependencies. Each node is associated with a probability function. Typically, the task of learning a Bayesian Network can be divided into two subtasks: initially, the DAG structure is formed; then its parameters are estimated. With regard to the general framework for creating Bayesian Networks, there are two possible scenarios. In the first, the structure of the network is given beforehand, for example, by an expert. In the second case, when the structure of network is unknown, several methods to learn the most accurate structure have been proposed in literature (see e.g. [5]). The parameter of this classifier, as in Weka, is:

- **D**: Use ADTree for learning.
  Default: false. *Range*: true or false.

1.1.2. **Naive Bayes:** The Naive Bayes classifier [3, 6, 7] is based on the so-called Bayesian theorem and is particularly suitable for high-dimensional datasets. Despite its apparent simplicity, this method can often outperform more sophisticated classification methods [8]. To classify new objects, the Bayes rule is applied to assign the membership of an object to a given class. Then the class displaying the highest posterior probability is chosen as the most likely class. This classifier is usually considered a naive approach because it assumes conditional independence and normal distribution for attributes in each class. Anyway, this classifier performs very well in many complex real-world situations [3]. A general discussion of the Naive Bayes method and its advantages are provided in Ref. [9]. The parameters of this classifier, according to Weka, are:

- **K**: use the kernel density estimator rather than normal distribution for numeric attributes.
  Default value: false. *Range*: true or false.

- **D**: use supervised discretization to convert numeric attributes to nominal ones.
  Default: false. *Range*: true or false.

1.2. Trees

A decision tree is a supervised automatic learning method that is employed for tackling both classification and regression tasks. It derives rules from a training set of objects represented by a number of attributes. The derived rules can be interpreted in a straightforward manner because they can be be visualized as a tree-like graph. An overview of decision tree methods is provided in Ref. [10]. In the current paper, the following algorithms were analyzed: C4.5, Random Forest and Simple CART.

1.2.1. **C4.5:** The C4.5 algorithm [11] derives from the Concept Learning System (CLS) and Iterative Dichotomiser 3 (ID3) algorithms [12, 13]. Given a set S of cases, the C4.5 first grows an initial tree using the divide-and-conquer algorithm as follows: if all the cases in S belong to the same class or S is small, the tree is a leaf labeled with the most frequent class in S. Otherwise, a test based on a single attribute with two or more outcomes is chosen. Then this test becomes the root of the tree with one branch for each outcome of the test, partitioning S into the corresponding subsets S_1, S_2 and so forth, according to the outcome for each case. Finally, the same procedure is applied recursively to each subset. There are usually many tests that can be chosen in this last step. The parameters present in the Weka implementation of the C4.5 are:

- **U**: whether pruning is performed.
  Default: false. *Range*: true or false.
- **S**: whether to consider the subtree raising operation when pruning.
  Default: true. *Range*: true or false.
- **A**: whether counts at leaves are smoothed based on Laplace.
  Default: false. *Range*: true or false.
- **C**: the confidence factor used for pruning (smaller values incur more pruning).
  Default: 0.25. $\{C \in \mathbb{R} \mid 0 < C \leq 0.5\}$.
- **M**: the minimum number of instances per leaf.
  Default: 2. *Range*: $\{M \in \mathbb{N} \mid M \geq 1\}$.
- **N**: determiner the amount of data used for reduced-error pruning.
  Default: 3. *Range*: $\{M \in \mathbb{N} \mid M \geq 2\}$.

1.2.2. **Random Forest:** The Random Forest classifier [14] is a set of classification trees. Each tree provides an indication about the class of the object. The class with the most votes is chosen as the most likely class. The method combines Breiman's bagging idea [15] with the random selection of features in order to construct a collection of decision trees with controlled variation. For each tree the training set is given by choosing n times with replacement from all N available training cases (i.e., the

method takes a bootstrap sample). For each node of the tree, m variables are randomly chosen from all M available features, which are then used for decision at that node. As an optional step, the tree obtained previously can be pruned using several strategies. Details concerning the implementation of this method can be found in Ref. [16]. The parameters of this classifier, as in Weka, are listed below.

- **I**: number of trees to build.
  Default: 10.
- **K**: number of features to consider.
  Default: 0. *Range*: $\{K \in \mathbb{N} \mid 0 \leq K \leq \text{dim}‡\}$.
- **depth**: the maximum depth of the tree.
  Default: 0. *Range*: $\{\text{depth} \in \mathbb{N}\}$.
- **S**: seed for random number generator.
  Default: 1.

1.2.3. **Simple Classification and Regression Tree (Simple CART):** The Simple CART method [17] is a modification of C4.5. In this algorithm, the decision represented in the tree may involve combinations of attributes, so the assumptions are no longer restricted to hyper-rectangular partitions. While C4.5 employs the gain ratio metric to rank tests, the Simple CART uses the Gini diversity index [18]. After the tree formation, a pruning step is applied via cost-complexity methods [19]. A pruning rule is applied to the split with the least contribution to the overall performance on the training data. Further details concerning CART methods can be found in Ref. [20, 21]. The involved parameters, according to Weka, are:

- **S**: random number seed.
  Default: 1.
- **C**: percentage of training data size.
  Default: 1. $0 < C \leq 1$. *Range*: $\{C \in \mathbb{R} \mid 0 \leq C \leq 1\}$
- **M**: the minimal number of instances at the terminal nodes.
  Default: 2.
- **N**: the number of folds used in the minimal cost-complexity pruning.
  Default: 5.
- **A**: use "1 SE" rule to make pruning decision.
  Default: false. *Range*: true or false.
- **H**: do not use the heuristic method for binary split.
  Default: false. *Range*: true or false.
- **U**: do not use the minimal cost-complexity pruning.
  Default: false. *Range*: true or false.

‡ dim is the number of attributes describing the dataset.

1.3. Lazy

Lazy (or instance-based) classifiers store all of the training samples and do not build a classifier until a new instance needs to be classified. It differs from eager classifiers that generalize the training data before receiving queries. Lazy-learning algorithms require less computation time during the training phase than eager-learning algorithms (such as decision trees, neural and Bayes nets). Nevertheless, instance-based classifiers demand more computation time during the classification process. A review of instance-based learning classifiers can be found in Ref. [22]. The lazy method studied here is the k-Nearest Neighbors, which is presented below.

1.3.1. **k-Nearest Neighbors (kNN):** The kNN method [23] is one of the simplest instance-based learning algorithms for supervised classification. The classification is based on the consensus among the classes of the nearest k neighbors of the unknown object. To classify an unlabeled object, initially the distance of the new object to the objects in the training dataset is computed. Then the k-nearest neighbors are identified and the most frequent class labels in the k-set used to determine the class label of the object through a voting process. According to Ref. [24], the k-Nearest Neighbors method tends to outperform SVM especially in multiclass classifications. The main parameters of the kNN, as in Weka, are:

- **K**: number of nearest neighbors used in classification.
  Default: 1. *Range*: $\{K \in \mathbb{N} \mid K \geq 1\}$.
- **I**: weight neighbors by the inverse or their distance.
  Default: false. *Range*: true or false.
- **F**: weight neighbors by 1 - their distance.
  Default: false. *Range*: true or false.
- **X**: select the number of nearest neighbors using hold-one-out evaluation on the training data.
  Default: false. *Range*: true or false.

1.4. Functions

This class of methods includes non-probabilistic classifiers, but unlike lazy classifiers, the system tries to generalize the training data before receiving queries. Most of the methods included in this family can be viewed as a straightforward application of optimization theory and statistical estimation. Three function algorithms are used in this paper: Logistic, Multilayer Perceptron and Support Vector Machines.

1.4.1. **Logistic:** The Logistic algorithm [6] uses the maximum likelihood rule for logistic (sigmoidal) posterior probabilities. It is a linear classifier and optimal for a family of different distributions. The idea behind it, as in many other statistical classification techniques, is to construct a linear predictor function that constructs a score from a

set of weights that are linearly combined with the explanatory variables (features) of a given observation using a dot product. The predicted outcome is that with the highest score. The difference between the multiclass Logistic model and other methods with the same basic setup (like linear discriminant analysis) is the procedure for determining (i.e. training) the optimal weights and the way in which the score is interpreted [25]. According to Weka, the parameters of this classifier are:

- **R**: set the ridge in the log-likelihood.
  Default: 10^{-8}. *Range*: $\{R \in \mathbb{R}\ \}$.
- **M**: set the maximum number of iterations.
  Default: -1. *Range*: $\{M \in \mathbb{N} \mid M \geq 1\}$.

1.4.2. **Multilayer Perceptron:** Neural networks are motivated by neuroscience, especially the McCulloch-Pitts' artificial neuron [26]. The Multilayer Perceptron [27] consists of multiple layers of nodes organized as a directed graph. It is a feed-forward artificial neural network model that maps the input dataset onto a set of appropriate outputs. The goal of the training process is to find the set of weight values that will cause the output from the neural network to match the actual target values as closely as possible. This task is done by an back-propagation training algorithm [28]. For classification tasks with categorical target variables, there are N neurons in the output layer producing N values, one for each of the N categories of the target variable. The parameters of this classifier, as described in Weka, are:

- **D**: learning rate decay will occur.
  Default: false. *Range*: true or false.
- **C**: normalizing a numeric class will not be done.
  Default: false. *Range*: true or false.
- **H**: the hidden layers to be created for the network.
  Default: 'a'. *Range*: {0,'a','i','o','t'}.
- **L**: learning rate for the back propagation.
  Default: 0.3. *Range*: $\{L \in \mathbb{R} \mid 0 \leq L \leq 1\}$.
- **M**: momentum rate for the back propagation.
  Default: 0.2. *Range*: $\{M \in \mathbb{R} \mid 0 \leq M \leq 1\}$.
- **N**: number of epochs to train through.
  Default: 500. *Range*: $\{N \in \mathbb{N} \mid N \geq 1\}$.
- **V**: percentage size of validation set to use to terminate training.
  Default: 0. *Range*: *Range*: $\{V \in \mathbb{N} \mid 0 \leq V \leq 100\}$.
- **E**: the consecutive number of errors allowed for validation testing before the network terminates.
  Default: 20. *Range*: $\{E \in \mathbb{N} \mid E \geq 1\}$.

1.4.3. **Support Vector Machine (SVM):** Currently support vector machines [29] are considered one of the most robust pattern recognition methods [18]. A SVM classifier is statistically based on the Vapnik-Chervonenkis (VC) dimension [27, 30] and assumes the soft margin hypothesis [30] (allowing mislabeled examples). It uses sequential minimal optimization (SMO) to solve the optimization problem (SMO breaks the problem into a series of smallest possible sub-problems, which are then solved analytically). The aim is to separate classes with hyperplanes. Geometrically, the margin corresponds to the shortest distance between the closest data points to a point on the hyperplane. For linearly inseparable data, some kernels have been proposed [31]. An efficient implementation is the core vector machine [32]. Because there are many such linear hyperplanes, the SVM guarantees that the function is found by maximizing the margin between the two classes. The involved parameters, as in Weka, are:

- **C**: set the parameter C of C-SVC, ϵ-SVR and ν-SVR
  Default: 1.
- **L**: tolerance parameter.
  Default: 0.001.
- **P**: set the ϵ in loss function of ϵ-SVR.
  Default: 0.1.
- **V**: turn off missing value replacement.
  Default: false. *Range*: true or false.
- **N**: set the parameter ν of ν-SVC, once-class SVM and ν-SVR.
  Default: 0.5.
- **E**: sets the exponent employed in the *polynomial kernel* $K(x, y) = \langle x, y\rangle^E$.
  Default: 1.0.
- **E**: sets the exponent employed in the *normalized polynomial kernel*:
  $$K(x, y) = \langle x, y\rangle / \sqrt{\langle x, x\rangle\langle x, y\rangle},$$
  where $\langle x, y\rangle = \text{PolyKernel}(x, y)$.
  Default: 2.0.
- **G**: sets the γ value employed in the *RBF kernel*:
  $$K(x, y) = \exp(-\gamma\langle x - y, x - y\rangle^2).$$
  Default: 0.01.
- **S**: sets the σ value employed in the *Pearson VII function-based universal kernel.*
  Default: 1.0.

2. Analysis with default parameters

Table S1. Statistics regarding the accuracy rate obtained in DB2F with classifiers set with the default values of parameters.

#	Classifier	Average (%)	Deviation (%)	Best case (%)	Worst case (%)
**1**	Naive Bayes	74.39	6.61	88.50	59.00
**2**	Logistic	72.67	6.67	86.25	58.75
**3**	Perceptron	72.67	6.38	85.50	59.50
**4**	C4.5	70.24	6.66	84.75	54.50
**5**	Simple CART	70.23	6.48	86.25	57.00
**6**	Bayes Net	68.68	7.00	85.25	54.75
**7**	Random Forest	69.94	6.80	83.25	53.75
**8**	kNN	68.67	7.49	82.50	51.00
**9**	SVM	67.44	6.62	82.75	52.00

Simple statistics summarizing the accuracy rate obtained in DB2F (dataset with two features) when all the parameters are set with their respective default values.

3. Statistical significance in the comparison of classifiers

Tables S3 displaying the statistical significance of the differences in performance. The database DB2F was evaluated with the default configuration of parameters.

Table S2. Statistics regarding the accuracy rate obtained in DB10F with classifiers set with the default values of parameters.

#	Classifier	Average (%)	Deviation (%)	Best case (%)	Worst case (%)
**1**	kNN	94.28	1.76	97.50	90.00
**2**	Perceptron	83.65	3.94	91.75	74.00
**3**	Random Forest	80.14	2.83	86.00	71.50
**4**	Naive Bayes	76.78	4.23	85.00	60.25
**5**	SVM	74.01	4.70	84.25	59.75
**6**	Logistic	71.16	4.69	80.25	59.00
**7**	Simple CART	71.07	4.71	80.25	59.00
**8**	C4.5	65.70	3.62	73.75	56.75
**9**	Bayes Net	56.87	5.16	67.00	41.25

Simple statistics regarding the accuracy rate obtained in DB10F when all the parameters of the classifiers are set with their respective default values.

Table S3. Information concerning the statistical significance of the difference between average accuracy rates considering the two-tailed t-student test under the homogeneity of variance hypothesis. The difference of average accuracy rates between two classifiers is marked with a • if they are significantly different.

	NBY	LOG	PRC	J48	SCA	BNT	RDF	kNN	SVM
NBY	–			•	•	•	•	•	•
LOG		–				•	•	•	•
PRC			–			•	•	•	•
J48	•			–					•
SCA	•				–				•
BNT	•	•	•			–			
RDF	•	•	•				–		
kNN	•	•	•					–	
SVM	•	•	•	•	•				–

4. One-dimensional analysis

Figure S1 shows the behavior of the accuracy in DB2F for all integer and real classifier parameters. Each dataset is represented by a different line. An interesting pattern refers to the oscillations in the accuracy along the neighborhood of the default parameter. Apart from the kNN classifier, the accuracy does not increase substantially when only one parameter varies. As for the C4.5, the accuracy rate seems to depend weakly upon the confidence factor (C). The variation of the other two parameters (M and N) above their default value seems to reduce the quality of the classification, as revealed by the decreasing behavior). With regard to the Logistic method, the value of the ridge in the log-likelihood does not affect the classifier, provided that it assumes a value below a given threshold. Whenever that threshold is exceeded, the quality of classification worsens considerably. Concerning the maximum number of iterations, the best choice is the default value. The Multilayer Perceptron classifier also seems to yield its best classification when parameters are set at their default values. Likewise the parameter R (the ridge in the log-likelihood) of the Logistic, when the parameters L (the learning rate for the back-propagation), M (momentum rate for the back-propagation), V (fraction of the validation set employed) and N (number of epoch to train through) take extreme values the quality of the classification decreases substantially. A similar behavior in DB10F can be observed in Figure S2.

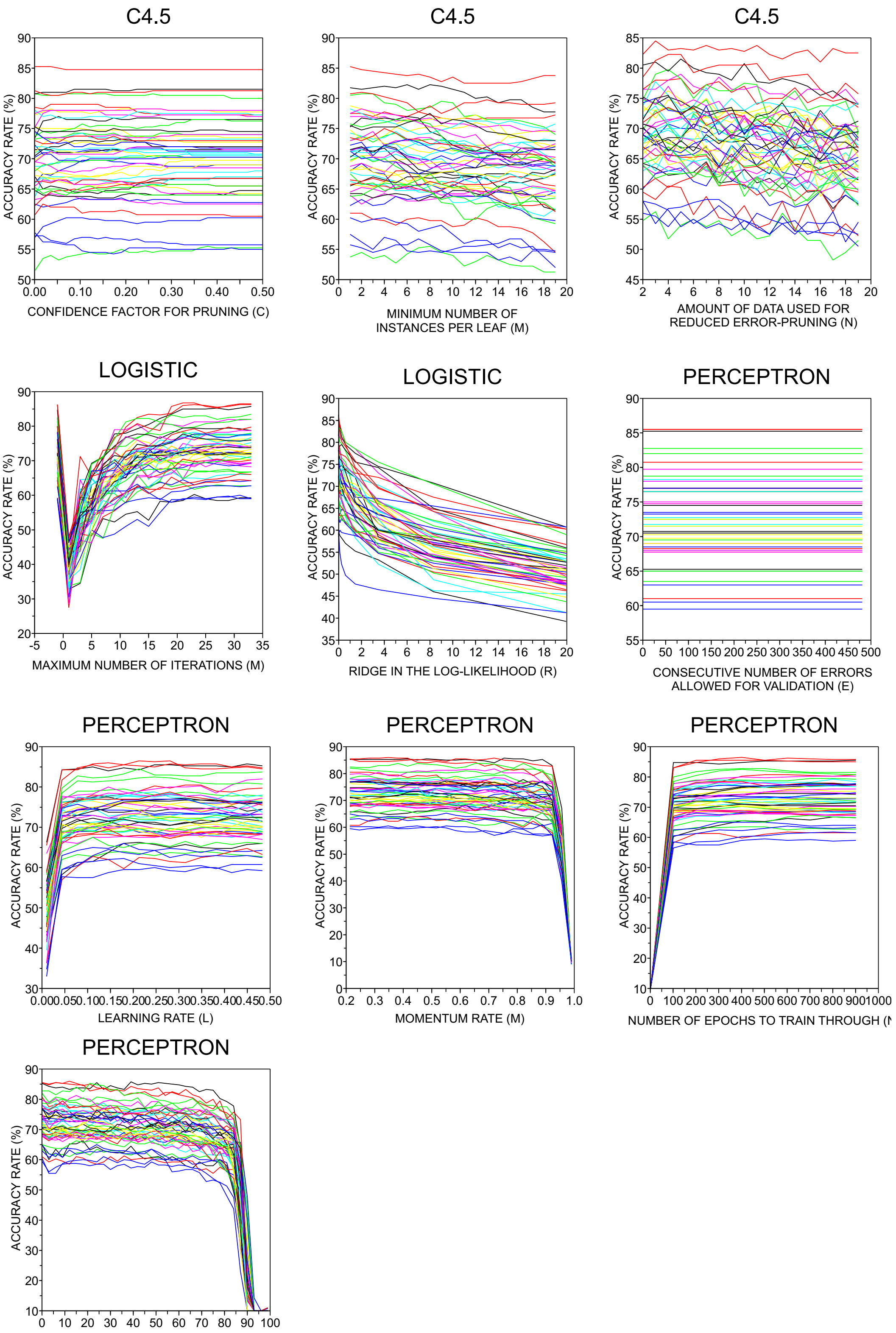

Figure S1. Variation of the accuracy rate when one varies a single parameter while keeping the remaining set at their default values in DB2F. Each line with the respective parameters represents the behavior of the accuracy rate concerning a single dataset. The default parameter is marked with a red vertical line. Note that, apart from the IBk classifier, all the classifiers usually achieve their best discriminability with the default parameters.

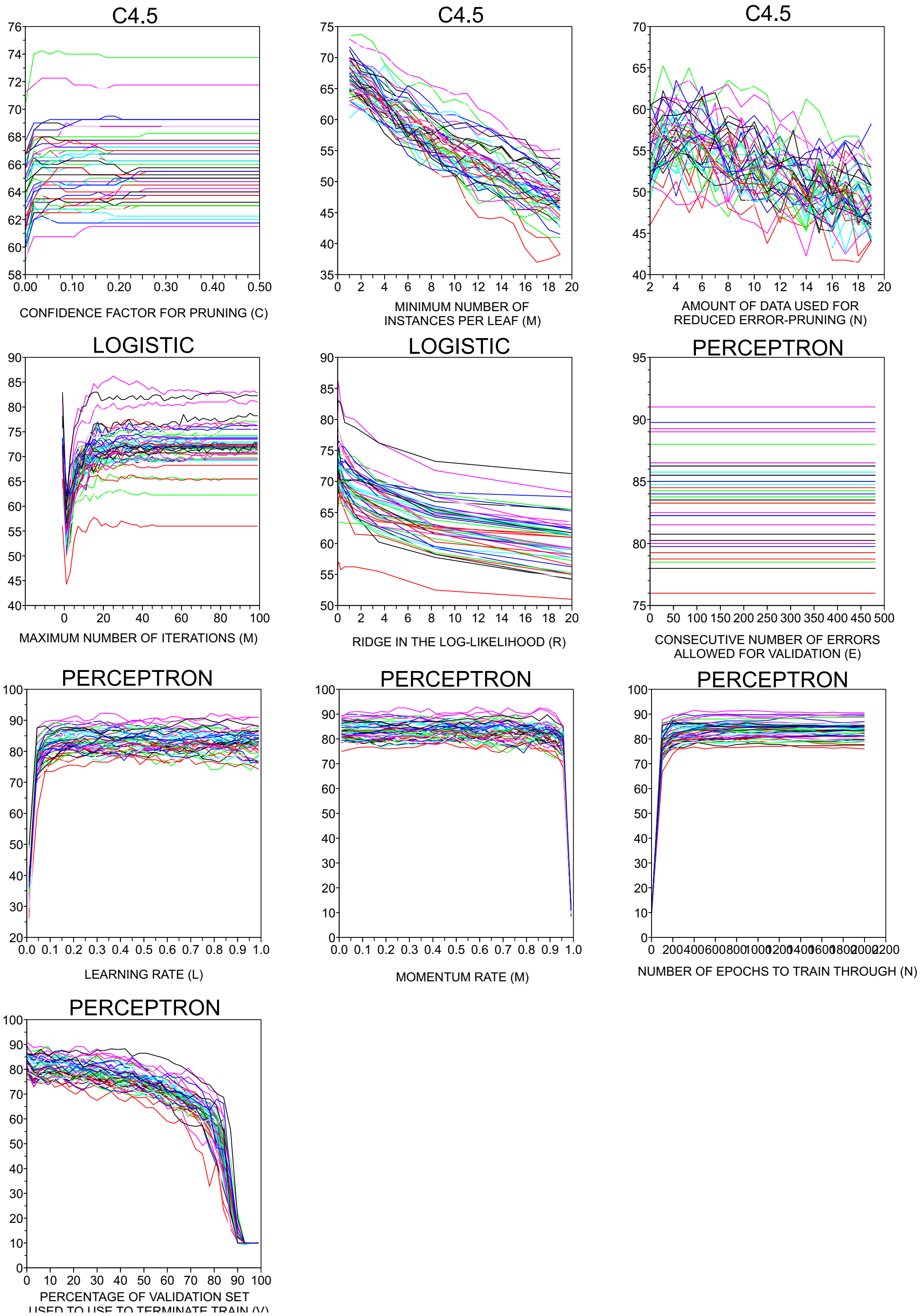

Figure S2. Variation of the accuracy rate when one varies a single parameter while keeping the remaining set at their default values in DB10F. Each line with the respective parameters represents the behavior of the accuracy rate concerning a single dataset. The default parameter is marked with a red vertical line. Note that, apart from the IBk classifier, all the classifiers usually achieve their best discriminability with the default parameters.

References

[1] Jain AK, Duin RPW, Mao J (2000) Statistical pattern recognition: a review. IEEE Trans. Pattern Analysis and Machine Intelligence 22: 4–37.

[2] Ian H, Frank E (2005) Data mining: pratical machine learning tools and techiniques. Morgan Kaufmann.

[3] Hand DJ, Yu K (2001) Idiot's Bayes: not so stupid after all? Statistical Review 69: 385–398.

[4] Pearl J (1988) Probabilistic reasoning in intelligent systems: networks of plausible inference. Morgan-Kaufmann Publishers.

[5] Heckerman D (1997) Bayesian networks for data mining. Data Min. Knowl. Discov. 1: 79–119.

[6] Mitchell T (1997) Machine Learning. McGraw-Hill.

[7] John G and Langley P (1995) Estimating continuous distributions in bayesian classifiers. Proceedings of the Eleventh Conference on Uncertainty in Artificial Intelligency.

[8] Huang J, Lu J, Ling CS (2003) Comparing naive Bayes, decision trees, and SVM with AUC and accuracy. IEEE International Conference on Data Mining : 553–556.

[9] Zhang H (2005) Exploring conditions for the optimality of Naïve Bayes. International Journal of Pattern Recognition and Artificial Intelligence 19: 183–198.

[10] Murthy SK (1998) Automatic construction of decision trees from data: a multi-disciplinary survey. Data Min. Knowl. Discov 2: 345–389.

[11] Quinlan JR (1993) C4.5: Programs for Machine Learning. Morgan Kaufmann.

[12] Hunt EB (1966) Experiments in induction. New York: Academic Press.

[13] Quinlan JR (1979) Discovering rules by induction from large collections of examples. Edinburgh University Press, 168–201.

[14] Breiman L (2011) Random Forests. Machine Learning 45: 5–32.

[15] Breiman L. (1996) Bagging Predictors. Machine Learning 24: 123–140.

[16] Criminisi A, Shotton J, Konukoglu E (2012) Decision forests: A unified framework for classification, regression, density estimation, manifold learning and semi-supervised learning. Foundations and Trends in Computer Graphics and Vision 7: 81–227.

[17] Breiman L, Friedman J, Olshen R, Stone C (1984) Classification and regression trees. Wadsworth International Group.

[18] Wu X, Kumar V, Quinlan JR, Ghosh J (2007) Top 10 algorithms in data mining. Springer-Verlag.

[19] Witten IH Frank E (2005) Data mining: practical machine learning tools and techniques. Morgan Kaufmann.

[20] Timofeev R (2004) Classification and regression trees (CART). Wirtschaftswissenschaftliche Fakultät.

[21] Tsoi AC, Pearson RA (1991) Comparison of three classification yechniques: CART, C4.5 and multi-layer perceptrons. Advances in Neural Information Processing Systems 3: 963–969.

[22] Mántaras RL, Armengol E (1998) Machine learning from examples: inductive and lazy methods. Data Knowl. Eng. 25: 99–123.

[23] Cover TM, Hart PE (1967) Nearest neighbor pattern classification. IEEE Trans. Information Theory 13: 21–27.

[24] Kuramochi M, Karypis G (2005) Gene classification using expression profiles: a feasibility study. International Journal on Artificial Intelligence Tools 14: 641–660.

[25] Xue J-H, Titterington DM (2008) Comment on "On discriminative vs. generative classifiers: A comparison of logistic regression and Naive Bayes". Neural Processing Letters 28: 169–187.

[26] McCulloch WS, Pitts W (1943) A logical calculus of the idea immanent in nervous activity. Bull. Math. Biophys. 5: 115–133.

[27] Haykin S (1999) Neural networks: a comprehensive introduction. Prentice Hall.

[28] Rumelhart D, Hinton G, Williams R. (1986) Learning representations by back-propagating errors. Nature 323: 533–536.

[29] Cortes C, Vapnik V (1995) Support-Vector networks. Machine Learning 20: 273.

[30] Vapnik V (1995) The nature of statistical learning theory. Springer Verlag.

[31] Schoelkopf C, Smola AJ (2002) Learning with kernels. The MIT Press.

[32] Tsang IW, Kwok JT, Cheung P-K (2005) Core vector machines: Fast SVM training on very large data sets. Journal of Machine Learning Research 6: 363–392.
